# Effects Of Internet-Based Tailored Advice on the Use of Cholesterol-Lowering Interventions: A Randomized Controlled Trial

**DOI:** 10.2196/jmir.1364

**Published:** 2010-09-13

**Authors:** Ruth Webster, Stephen CH Li, David R Sullivan, Kathy Jayne, Steve YS Su, Bruce Neal

**Affiliations:** ^4^Clinical Trials Service UnitUniversity of OxfordOxfordUnited Kingdom; ^3^Department of Clinical BiochemistryRoyal Prince Alfred HospitalSydneyAustralia; ^2^Institute of Clinical Pathology and Medical ResearchWestmead HospitalSydneyAustralia; ^1^The George Institute for International HealthSydneyAustralia

**Keywords:** Heart disease, cholesterol, computers, Internet, World Wide Web, randomized trial

## Abstract

**Background:**

Elevated low-density lipoprotein (LDL) cholesterol is a leading risk factor for cardiovascular disease. Despite the availability of proven interventions to lower LDL cholesterol, their use remains subobtimal. Many websites provide interactive, tailored advice on cardiovascular risk in an attempt to help bridge this evidence-practice gap, yet there is little evidence that provision of such a tool is effective in changing practice.

**Objectives:**

The objective was to define the effects on use of cholesterol-lowering interventions of a consumer-targeted tailored advice website.

**Methods:**

This was a prospective, double-blind, randomized controlled trial open to any adult Australian with access to the Internet. A total of 2099 participants were randomized. Of these, 45% were male, the mean age of all participants was 56, and 1385 (66%) self-reported hypercholesterolemia. Follow-up information was obtained for 1945 (93%). Participants completed a brief online questionnaire. Individuals assigned to intervention received immediate, fully automated, personally tailored advice (based on current guidelines) regarding the need for commencement of statin therapy, increased statin therapy in those already on treatment, and nondrug intervention strategies. Control group participants were directed to static Web pages providing general information about cholesterol management.

**Results:**

The primary outcome was the proportion of participants that commenced or increased use of prescribed cholesterol-lowering therapy. Of the total 2099 randomized participants, 304 (14%) met eligibility criteria for cholesterol-lowering therapy but were not prescribed treatment, and 254 (12%) were prescribed treatment but were not achieving the recommended target level. Treatment was commenced or increased in 64 (6.0%) of the 1062 intervention group participants and 79 (7.6%) of the 1037 control group participants (% difference = -1.6%, 95% confidence interval [CI] -3.75 to 0.57, *P* = .15). No differences were found between the randomized groups for the secondary outcomes of “discussed treatment with a health professional” (% difference = -3.8%, 95% confidence interval [CI] -8.16 to 0.19, *P* = .08), “had their cholesterol checked” (% difference = -1.5%, 95% CI -5.79 to 2.71, *P* = .48), “had their blood pressure checked” (% difference = 1.4%, 95% CI -2.55 to 5.34, *P* = .49) or made a lifestyle change (*P* values between .49 and .96).

**Conclusions:**

Despite providing specific carefully tailored advice, this website had no detectable effect on cholesterol management strategies. This finding raises considerable uncertainty about the value of Internet-based tools providing tailored advice directly to consumers.

**Trial Registration:**

NCT00220974; http://clinicaltrials.gov/ct2/show/NCT00220974 (Archived by WebCite at http://www.webcitation.org/5sdq63rrY)

## Introduction

An elevated level of low-density lipoprotein (LDL) cholesterol is one of the leading risk factors for cardiovascular disease and has been targeted by the lipid management guidelines of many organizations worldwide [1-3]. Despite proven cost-effectiveness [4,5], the use of cholesterol-lowering interventions remains suboptimal, with many eligible individuals untreated and many of those treated failing to reach the recommended target levels [6-8].

Research has shown that tailored information is more effective than generic communications in influencing health behaviors [9]. Delivery of health information via computer has also been shown to increase efficacy [10]. Widespread community access to the Internet [11] and increasing use of the Internet as a source of information about health [12] provide a novel opportunity for low-cost, Internet-based, community-mediated health care delivery. Previous studies have shown that highly interactive health communications applications that allow multiple interactions with participants over time may have a positive effect on knowledge and social support. In addition, there is some evidence that use of these applications results in improved behavioral and clinical outcomes in people with chronic disease [13]. There is also some evidence that such applications can positively influence behaviors related to cardiovascular risk such as nutrition and physical activity [14].

In the field of cardiovascular prevention, providing tailored behavior change messages has been shown to enhance uptake of information compared with simple provision of health risk information [15], with some websites offering such tailored advice directed at the consumer [16-17]. These websites tend to be simple, without the highly interactive features that have been shown to be effective in changing behavior. If shown to be effective, these simpler websites may help bridge the evidence-practice gap in cardiovascular disease prevention in a more cost-effective manner than larger, more complex Web-based interventions. Studies of simpler, Internet-based applications providing tailored advice in real-life settings have varied in size and effectiveness with more recent, larger studies showing promise [18-21]. At the time of our trial, there were few data that precisely and reliably defined the impact of websites offering simple tailored advice on objective outcomes.

In an attempt to address this research gap, we report here the results of the Internet-based Cholesterol Assessment Trial (I-CAT), which sought to define the effects on cholesterol management of a simple, real-life consumer-mediated website offering tailored advice typical of those available on the Internet. The website provided individually tailored advice to adult Australians about their need for cholesterol-lowering treatment according to established Australian guidelines [22-23].

The primary aim of this trial was to determine the effects on the use of prescribed cholesterol-lowering treatment of access to a website that provided fully automated, individually tailored advice about eligibility for cholesterol-lowering treatment according to established national guidelines. This was compared against access to typical static Web pages. The null hypothesis of no effect of the intervention compared to control on the primary outcome was tested. The secondary aim of the trial was to evaluate whether it was possible to improve the cholesterol management of the friends and relatives of the index participants as measured by the same outcomes.

## Methods

The I-CAT was a double-blinded, randomized, controlled trial conducted from October 6, 2004, through July 5, 2006. The trial was approved by the Human Ethics Committee of the University of Sydney with all participants providing informed consent. Aside from mail and telephone contact with initial nonresponders to follow-up, the study was done entirely through a secure website established for the trial with access for participants achieved through use of unique individual usernames and passwords.

### Participants and Recruitment

The trial was open to all adults aged 18 years or over resident in Australia, although recruitment strategies were targeted toward individuals likely to require cholesterol-lowering therapy by focusing recruitment initiatives on health care facilities and seniors’ organizations. Recruitment was achieved by using a range of approaches including posters, printed and electronic invitations, website links, radio broadcasts, newspaper advertisements, and direct referrals to the study website by health care providers. Potential participants were required to read an online participant information sheet and complete an online consent form. Participants were not informed of the precise randomized comparison being made and were simply told that they were participating in a trial that sought to “find out if advice about cholesterol provided on the Internet can improve your cholesterol management.” If participants were randomized to the active arm, they were immediately asked to refer their friends and relatives to the website in order to determine whether or not the website was able to gain access to networks of friends and relatives via personal referral and thus influence the health behavior of friends and relatives as well. Control group participants were requested to do the same only after completing follow-up for the primary and secondary outcomes at which time they received individualized advice. Friends or relatives who responded to referral from a randomized participant were not randomized; they were simply asked to provide informed consent, asked to complete the baseline and 8 week follow-up questionnaires, and were then documented as a referral from a randomized participant for the purposes of outcome evaluation. Information was collected from the referred participant in an effort to link them back to the referrer in order to determine any differences in referral patterns between intervention and control participants.

### Baseline Data Collection

The baseline questionnaire was administered to all consenting participants and sought demographic details, cardiovascular disease history, risk factors, cholesterol levels, use of any medications or other strategies to reduce their cholesterol-related cardiovascular risk, and any family history of cardiovascular disease or high cholesterol levels (see Multimedia Appendix 1). The information sought was sufficient to define (1) eligibility for statin therapy according to the February 2004 Australian Prescriber Benefit Scheme criteria [22]; (2) the need for increased statin therapy to achieve the lipid targets recommended by the Cardiac Society of Australia and New Zealand guidelines [23]; (3) the need for commencement of nondrug intervention strategies that might be used to lower their risk of a cholesterol-related event (including recommending weight loss if their body mass index (BMI) was greater than 24 and they were not trying to lose weight; taking regular exercise; starting a healthy diet; and, if they were not already doing so, using cholesterol-lowering margarine if they had been diagnosed with high cholesterol or were indicated for cholesterol-lowering treatment [24]); and (4) the likelihood of there being a familial tendency to cholesterol-related disease (if their total cholesterol > 9 mmol/L, they were of aboriginal heritage, or they had been diagnosed with genetic hypercholesterolemia or a had a family history of premature heart disease and previous diagnosis of high cholesterol). The questions were simple to answer and took no more than a few minutes to complete. Individuals were not required to be able to answer all questions to proceed to randomization, but it was made clear that the more information that was provided the better.

### Randomization

Randomization followed immediately after baseline data collection. Randomization was done automatically in real time by a central computerized service run by the investigators at The George Institute for International Health. There was no stratification since the large number of participants would ensure reasonable balance of characteristics between randomized groups. Investigators were blinded to the allocation of all individuals throughout the trial. This was true both for the collection of data by the automated email prompts and for the data collected by phone or mail. Unblinding only occurred once follow-up of all participants was complete.

### Intervention and Control

The intervention group received immediate personally tailored cholesterol management advice from a fully automated computer algorithm that used the information collected in the baseline questionnaire. If data were missing from the questionnaire, the response was assumed to be negative except for responses to the question about lipid levels. If lipid levels were absent, the tailored advice was qualified and participants were informed that full assessment of their situation was not possible without lipid values and that it would be helpful if they had their cholesterol measured. The computer algorithm was tested using multiple hypothetical scenarios to ensure that the advice given was accurate.

**Figure 1:**
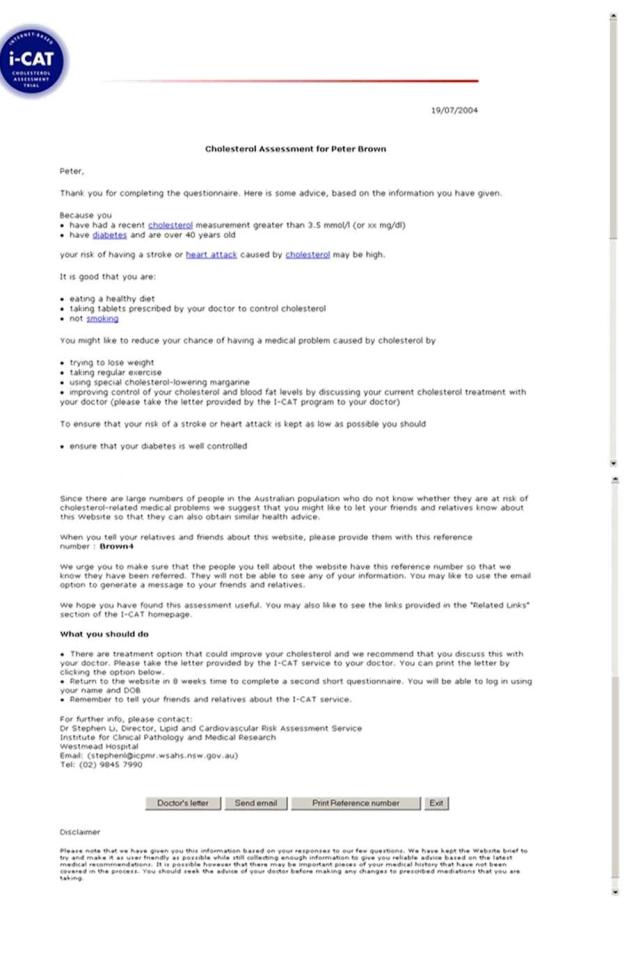
Screenshot of sample tailored advice

The advice provided comprised specific recommendations about the need for commencement of statin therapy, increased statin therapy in those already on treatment, and nondrug intervention strategies. Based on each individual’s likelihood of a family history of cholesterol-related disease, participants were also advised that their relatives could also benefit from visiting the site. Participants could print each recommendation and the associated reasons for that recommendation in the form of an automatically generated individually tailored letter that could be taken to their doctor. Participants could also print off information that might be suitable for passing on to friends or relatives or directly email the website details to these people. The information was badged with the logos of The George Institute for International Health, the University of Sydney, Western Sydney Area Health Service, and the Institute of Clinical Pathology and Medical Research, and it was made clear that the advice provided was based upon the recommendations of the 2004 Pharmaceutical Benefit Scheme Lipid Management Guidelines and the 2001 Australian and New Zealand Lipid Management Guidelines. The credibility of the output was further enhanced by the advice being electronically signed by one of the authors (SL) who was identified as the Director of the Lipid and Cardiovascular Risk Assessment Service, Westmead Hospital. An example of the tailored advice is shown in Figure 1. In addition to the tailored advice, participants were referred to links on the website containing generic information on cholesterol.

Participants in the control group were provided with only general information about lowering cholesterol in the form of links to relevant sites containing static Web pages, but control group participants were given no specific management recommendations. Control group participants, however, were provided with 35 links to fact sheets and information including pages from the National Heart Foundation, the Australian Department of Health and Aging, the NSW Department of Sport and Recreation and many others (all links can be viewed on the website, www.cholesterolcheck.info) [25].

All relatives and friends that were referred to the website, irrespective of whether they were referred by an individual assigned to the intervention or the control group, received immediate personally tailored cholesterol management advice from the computer algorithm. The control group participants were not specifically asked to refer friends or relatives until after they had completed follow-up to reduce the risk of contamination. (For example, if the friend or relative showed the control group participant the specific advice that was generated, this may have impacted the control group participant’s decision and/or timing in returning to the website to complete the next questionnaire.)

### Follow-up

All randomized participants were scheduled for follow-up 8 weeks after randomization. Follow-up comprised an email reminder to log on and complete an online questionnaire seeking information about each of the study outcomes. The follow-up questions were phrased such that they were simple to answer and designed such that the entire follow-up questionnaire would take no more than a few minutes to complete (see Multimedia Appendix 2). Participants that failed to return to the website after initial email prompting (including friends and relatives) were contacted successively by mail and phone to achieve follow-up data collection. Participants in the control group received their individually tailored advice after completing the second questionnaire. All participants were invited to fill in a feedback questionnaire at the completion of the study in order to ascertain their views on the usefulness of the website and the information provided.

### Study Outcomes

The primary outcome was the number of participants that reported commencing or increasing treatment with lipid lowering medication. Secondary outcomes were the number of participants that had their cholesterol levels checked, visited a doctor, commenced eating a healthy diet, started trying to lose weight, started taking regular exercise, started using special cholesterol-lowering margarine, stopped smoking, had their blood pressure checked, or recommended the website to a friend or relative.

### Adverse Events

No significant adverse events were anticipated as a result of our intervention although some participants potentially may have been falsely reassured of being at low risk of vascular disease by our tailored advice if they did not provide full details of their clinical situation. This issue was addressed by carefully advising participants that our intervention may not take into account their full medical history and that they should discuss their results with their regular doctor. Participants were not specifically asked if they had experienced any adverse effects as a result of our study.

### Statistics

#### Power

The trial was initially planned to recruit 3938 individuals to achieve 90% power (alpha = .05) to detect a 2.5% or greater difference between randomized groups in the proportion reporting the primary outcome. This estimate assumed that the primary outcome event rate in the control group would be about 5% and that it would be increased by a half, to about 7.5% in the intervention group. The trial actually randomized 2099 individuals with an event rate of 7.6% in the control group, and this provided 90% power (alpha = .05) to detect a 4% or greater absolute difference between randomized groups in the proportion reporting the primary outcome. The power calculations were carried out with PASS 2008 software (NCSS Statistical and Power Analysis Software, Kaysville, UT, USA) using Mantel-Haenszel, likelihood ratio, and *z* tests.

#### Analysis

Statistical analyses were done with SAS version 9.1 (SAS Institute Inc, Cary, NC, USA). Characteristics of participants were summarized as proportions or means with standard deviations. Comparisons of proportions were done using Pearson’s chi-square test without continuity correction. Estimates of effect size for the outcomes summarized as proportions are presented as differences in proportions and 95% confidence intervals. For these analyses the denominators were the total number randomized in each group such that wherever possible, estimates were unbiased “intention-to-treat” analyses. Comparisons between the mean values of continuous outcome measures were made using *t* tests, and estimates of effect sizes were presented as mean differences with 95% confidence intervals. These latter analyses included only those individuals for whom the data were not missing. All tests were two-sided and univariate, missing values were not imputed, and a *P* value of .05 or less was interpreted as unlikely to have arisen by chance.

## Results

### Recruitment and Follow-up

**Figure 2:**
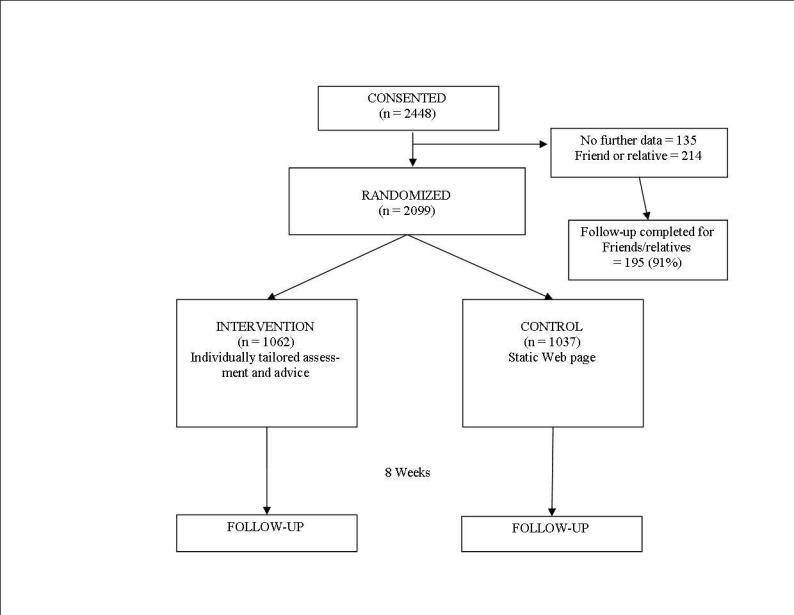
Flow chart

In total, 2448 individuals consented to participate comprising 2099 randomized participants, 214 friends or relatives referred by randomized participants, and 135 individuals that consented but provided no further data and were not randomized (Figure 2). Recruitment was terminated prior to the target recruitment number due to the prolonged time taken to recruit participants. Of the total number of randomized participants, 1062 were assigned to intervention and 1037 to control; 93% of the total provided follow-up data with 750 (36%) requiring mail and/or telephone follow-up to achieve this. Follow-up data was collected at a median of 10 weeks (range 7 to 42 weeks) after randomization with the active group taking slightly longer to respond than the control group (median 10.3 vs 10.1 weeks).

### Participant Characteristics

Trial participants were an average of 56 years old and 918 out of 2099 (44%) were male (Table 1). About two-thirds (1387/2099) had a self-reported prior diagnosis of hypercholesterolemia, one-third (689/2099), a family history of premature coronary heart disease, and one in ten (241/2099) had been told that they or a family member had a possible heritable component to their elevated cholesterol levels. Most participants reported that they were on a healthy diet and taking regular exercise, although nearly two-thirds (1258/2099) responded that they were trying to lose weight. The overall mean body mass index (BMI) of participants was 26.7kg/m^2^. The baseline characteristics of the intervention and control groups were generally well balanced—the exception to this was that the intervention group had a slightly greater likelihood of having family members at high risk of hypercholesterolemia.

**Table 1:** Baseline characteristics of randomized participants

		Number with Data	Intervention (n = 1062)	Control (n = 1037)
Male		2099	482 (45)	436 (42)
**Medical history**				
	High cholesterol, n (%)	2098	710 (67)	677 (65)
	Hypertension, n (%)	2086	364 (34)	390 (38)
	Diabetes mellitus, n (%)	2099	129 (12)	123 (12)
	Coronary heart disease, n (%)	2098	94 (9)	84 (8)
	Peripheral vascular disease, n (%)	2097	26 (2)	21 (2)
	Cerebrovascular disease, n (%)	2099	32 (3)	37 (4)
	Familial hypercholesterolemia, n (%)	2093	34 (3)	33 (3)
	Participant or family has been told they have genetic hypercholesterolemia, n (%)	2095	111 (10)	130 (13)
	Family history of premature coronary heart disease, n (%)	2098	331 (31)	358 (35)
**Lifestyle factors**				
	Current smoker, n (%)	2098	75 (7.1)	77 (7.4)
	Eating a health diet, n (%)	2086	892 (84)	862 (83)
	Trying to lose weight, n (%)	2090	620 (58)	638 (62)
	Taking regular exercise, n (%)	2088	770 (73)	766 (74)
	Using cholesterol-lowering margarine, n (%)	2091	339 (32)	325 (31)
	Currently on lipid modifying treatment, n (%)	2088	295 (28)	266 (26)
	Awareness of own lipid subfractions, n (%)	2057	309 (29)	274 (26)
	Not on medication and indicated for treatment^a^, n (%)	2024	160 (15)	144 (14)
	On medication but not reaching target, n (%)	2076	141 (13)	113 (11)
	Family at risk^b^, n (%)	2021	330 (31)	239 (23)
	Age (years), mean ± SD (n)	2099	55.8 ± 12.21 (1062)	56.3 ± 11.88 (1037)
	Weight (kg), mean ± SD (n)	2074	77.3 ± 17.74 (1049)	76.5 ± 16.54 (1025
	Height (cm), mean ± SD (n)	2075	170 ± 9.32 (1053)	169.1 ± 9.46 (1022)
	BMI (kg/m2), mean ± SD (n)	2054	26.7 ± 5.34 (1042)	26.6 ± 4.93 (1012)
	Highest total cholesterol (mmol/L), mean ± SD (n)	1139	6.7 ± 1.41 (577)	6.9 ± 1.85 (562)
	Recent total cholesterol (mmol/L), mean ± SD (n)	1196	5.6 ± 1.25 (614)	5.7 ± 1.28 (582)
	Recent LDL cholesterol (mmol/L), mean ± SD (n)	535	3.5 ± 1.20 (285)	3.5 ± 1.15 (250)
	Recent HDL^c^ cholesterol (mmol/L), mean ± SD (n)	551	1.7 ± 0.75 (296)	1.7 ± 0.68 (255)
	Recent triglyceride (mmol/L), mean ± SD (n)	539	1.6 ± 1.05 (277)	1.7 ± 1.71 (262)

^a^ Calculation based on lipid guidelines and Pharmaceutical Benefit Scheme guidelines current at time of trial

^b^ Calculation based on participant answers indicating possible high risk of hypercholesterolemia among family members

^c^ High density lipoprotein

### Eligibility for Starting or Increasing Cholesterol Lowering Treatment

Based on our algorithm, among the 2099 randomized participants there were 561 (27%) that were using prescribed treatment of which 254 were not meeting recommended targets and were deemed eligible for increased treatment. Of those not on treatment, 20% (304/1538) met the criteria for treatment.

### Effects of Intervention Compared With Control

Tables 2 and 3 show the results for the primary and secondary outcomes for the main study aim. The primary outcome, commencement or increase in cholesterol-lowering therapy, was observed in 6.8% (143/2099) of the randomized participants, 6.0% (64/1062) in the intervention group and 7.6% (79/1037) in the control group (% difference = -1.6, 95% confidence interval [CI] -3.75 to 0.57). For new treatment, the percent difference was -1.4% (95% CI -2.87 to 0.23), and for increased treatment, it was -0.3% (95% CI -1.83 to 1.29).

**Table 2:** Effects of treatment on primary and secondary outcomes (binary) among randomized participants

		Intervention (n = 1062)	Control (n = 1037)	% Difference (95% CI)	*P* value
Primary outcome		n (%)	n (%)		
**Commenced or increased cholesterol-lowering therapy**		64 (6.0)	79 (7.6)	-1.6 (-3.75 to 0.57)	.15
	Commenced treatment	29 (2.7)	42 (4.1)	-1.4 (-2.87 to 0.23)	.09
	Increased treatment	35 (3.3)	37 (3.6)	-0.3 (-1.83 to 1.29)	.73
**Secondary outcomes (binary)**					
	Discussed treatment with a health professional	521 (49.1)	549 (52.9)	-3.8 (-8.16 to 0.19)	.08
	Had blood cholesterol checked	465 (43.8)	470 (45.3)	-1.5 (-5.79 to 2.71)	.48
	Commenced eating a healthy diet	85 (8.0)	86 (8.3)	-0.3 (-2.63 to 2.05)	.81
	Commenced trying to lose weight	107 (10.1)	103 (9.9)	0.2 (-2.42 to 2.71)	.91
	Commenced taking regular exercise	112 (10.5	100 (9.6)	0.9 (-1.67 to 3.50)	.49
	Commenced using cholesterol-lowering margarine	112 (10.5)	105 (10.1)	0.4 (-2.18 to 3.03)	.75
	Stopped smoking	9 (0.8)	9 (0.9)	-0.1 (-0.81 to 0.77)	.96
	Blood pressure checked	744 (70.1)	712 (68.7)	1.4 (-2.55 to 5.34)	.49
	Referred friend or relative to the website	176 (16.6)	91 (8.8)	7.8 (4.97-10.62)	<.001
	Number of friends or relatives that visited the website	69 (7)	23 (2)	3.1 (1.9-5.0)	<.001

**Table 3:** Effects of treatment on secondary outcomes (continuous) among randomized participants

Secondary Outcomes (Continuous)	Mean ± SD	Mean ± SD	Mean Difference (95% CI)	*P* value
Recent total cholesterol (mmol/L)	5.45 ± 1.21 (n=600)	5.51 ± 1.23 (n=593)	-0.07 (-0.21 to 0.07)	.34
Recent low-density lipoprotein (LDL) cholesterol (mmol/L)	3.38 ± 1.13 (n=317)	3.31 ± 1.06 (n=306)	0.07 (-0.1 to 0.24)	.43
Recent high-density lipoprotein (HDL) cholesterol (mmol/L)	1.65 ± 0.69 (n=330)	1.67 ± 0.67 (n=314)	-0.03 (-0.14 to 0.08)	.59
Recent triglyceride (mmol/L)	1.71 ± 1.29 (n=323)	1.62 ±1.59 (n=312)	0.10 (-0.13 to 0.32)	.40
Current weight (kg)	77 ± 17.2 (n=937)	77 ± 16.74 (n=926)	0.03 (-1.51 to 1.57)	.97

In regard to the secondary outcomes, there were no significant differences between randomized groups in the proportions that visited a doctor, had their cholesterol levels checked, commenced eating a healthy diet, started trying to lose weight, started taking regular exercise, started using cholesterol-lowering margarine, stopped smoking, or had their blood pressure checked (all *P* > .08) (Table 2). The one exception was that more individuals in the intervention group (176/1062, 17%) than the control group (91/1037, 9%) referred one or more friends or relatives to the website (% difference = 7.8 %, 95% CI 4.97-10.62, *P* < .001). These referrals resulted in 69 friends or relatives of the intervention group participants and 23 friends or relatives of the control group participants actually visiting the website within the follow-up period (ie, prior to the index case returning to complete the second questionnaire). The population visiting the website within this time frame was used to calculate the outcomes for friends and relatives, as only intervention group participants were specifically asked to refer friends and relatives to the website after the first questionnaire, whereas the control group received this request after completing the second questionnaire. In addition, 92 other friends and relatives that visited the website did not provide sufficient information to link them back to the referring index participant. Of the 69 friends or relatives of intervention participants, 5 commenced or increased cholesterol-lowering therapy compared with none of the friends or relatives of the control group participants (0.5% vs 0%; *P* = .06). Significantly more of the intervention group participants had friends or relatives discuss treatment with a health professional (26/1062, 2% vs 5/1037, 0.5%, *P* < .001), had their cholesterol checked (26/1062, 2% vs 6/1037, 0.6%; *P* < .001), commenced cholesterol-lowering margarine (6/1062, 0.6% vs 0/1037; *P* = .03), were checked for diabetes (19/1062, 2% vs 8/1037, 0.8%; *P* = .04) or had their blood pressure checked (40/1062, 4% vs 16/1037, 2%; *P* = .002) after receiving individualized advice from the website, but otherwise there were no differences for the other outcomes (all *P* > .09).

In total, 1144 of the 2448 (47%) participants provided written feedback about one or other aspect of the study. The study was generally perceived as providing useful (926/1144, 81%), trustworthy (857/1119, 95%) and clear (1027/1126, 91%) information. Important issues that arose in the “free text” section were around the issues of participants not understanding cholesterol levels or not having been given test results and so being unsure of how to respond to those questions. In addition, a third (51/156, 33%) of those that put a “free text” comment remarked on the inappropriateness of the questionnaire for their particular circumstances.

### Adverse Events

To our knowledge, no adverse events occurred during the course of the study.

## Discussion

Our intervention provided consumers with individualized user-friendly advice from a credible source but had no detectable impact on any important aspect of participant treatment or participant behavior related to cholesterol management. This finding raises doubt about the value of the multitude of consumer-targeted websites that seek to improve participant health and medical care using this type of simplified approach.

There are many possible reasons why there was no clear effect of the intervention in our study. First, achievement of change in treatment required the successful completion of a sequence of events. Specifically, the participant had to decide to act upon the advice received from the website, print out the materials provided, make an appointment to visit their doctor, and then attend the visit. The stages of change model outlines a complex cycle that takes place in order to change behavior [26]. A website offering tailored advice is only likely to affect people who are already motivated to change and are on the brink of taking action and therefore targets people at one point of the change cycle only. Furthermore, recent large-scale trial data and meta-analysis has confirmed that increased depth of tailoring and use of multiple behavioral change techniques based on established models of behavioral change are more effective [21,27]. Based on this evidence as well as our trial outcome, there can be little justification for expecting significant effects on clinical outcomes from simple tailored advice websites. It is likely that health websites must provide more comprehensive support to help users achieve the changes in behavior sought, that is, health websites must provide a more highly interactive tool or one that provides an additional resource.

If the participant did attend his or her general practitioner’s clinic, the doctor then had to be persuaded by the advice and commence or increase cholesterol-lowering treatment accordingly. There are multiple points at which this chain of events could break down, and there is evidence from other sources to suggest why this may not occur [28].

Another issue of primary importance is the willingness of physicians to respond to information provided to them by their patients. While doctors frequently use computerized systems and the Internet to seek information, they may be less likely to act upon material they receive indirectly from the Internet via their patients [29]. That said, the credibility of the information provided by the website did not appear to be a major issue, with the majority of participants that gave feedback about visiting their doctor indicating that both they and their physicians viewed the website outputs as useful and trustworthy.

A further consideration is that the study was relatively short, providing a limited time frame during which change in treatment had to occur. In retrospect, it may have been too brief a period to make treatment changes for some participants although it is of note that there were no effects of the intervention on actions intermediate to treatment change. For example, the number of participants making visits to the doctor or having assays of blood lipids was not greater in the intervention compared with the control group and could reasonably have been expected to be increased in the time period available.

It is also possible that cholesterol management was a more difficult management problem among our study participants as compared with the general population at risk of cholesterol-related disease. Individuals prepared to seek out solutions on the Internet may previously have been through multiple other efforts to control their lipid levels; the website may not have had much new to offer, and this could have reduced the potential for the intervention to impact upon treatment. There was some participant feedback to indicate that this was the case, with some respondents reporting that they found the website to be inappropriate to their situation because they had already tried most of the suggested interventions, had side effects to suggested treatment, or had high levels of cholesterol that were resistant to usual therapy. The approach used by the I-CAT website, which was to address the usual cholesterol problems with the usual cholesterol-lowering solutions, may not be appropriate for the real-world setting of health websites because it may actually be the more unusual cases that comprise a large proportion of website users.

An alternate interpretation of the study findings might be that while the tailoring component of the website intervention we evaluated was not important, the large numbers of participants across the two groups that reported taking some action suggest that the website was producing some effect. It is possible that simply completing the risk factor questionnaires was sufficient to drive people to consult a health care provider or have their cholesterol checked even if tailored advice was not provided. However, a more likely explanation is that the individuals that enrolled in this study were a self-selected and highly motivated group that would have taken these actions irrespective of anything they did as part of the trial. It is also possible that a ceiling effect had been reached for some of the recommendations, with significant numbers already reporting some actions at the baseline assessment. Clearly this study cannot reliably address the question of whether simply accessing static Web pages can change health-related behaviors, although it is generally agreed that simply viewing general information is not an effective means of achieving individual behavioral change [30].

Contamination of our control arm could have occurred if some family members in a given household were randomized to the control group and some to the intervention group. This would have had the effect that participants in the control arm would then have been aware that personalized information was provided to other participants and may have influenced them to return to the website for follow-up more promptly than other control group participants. In relation to the primary outcome, we believe it is unlikely to have importantly influenced the results as the advice was specifically tailored to the recipient and was not generalizable to other participants. In addition, rather few control group participants had friends or relatives visit the website.

The finding that the intervention group referred more friends or relatives to the website compared with control participants was the one positive result from the trial and might be of some value. The targeted identification and referral of individuals in this way might, for example, be a method that could be used as part of genetic cascade follow-up programs for conditions such as familial hypercholesterolemia [31].

The chief strength of this study was its large scale, robust randomized design, and real-world evaluation of the intervention under investigation. There has been no prior study that begins to approach I-CAT in regard to this combination of characteristics, and on this basis, the data presented here represent a major advancement of knowledge. However, the study also had some shortcomings. First, while the study was large, there were challenges with recruitment, and the original recruitment target was not met. It is possible, therefore, that the study failed to detect a small real effect of the intervention on the primary outcome. That said, the absence of any effect on intermediate outcomes for which there was much better statistical power suggests that this is probably not the case. Second, the study relied upon participant-reported data both at baseline and follow-up and in many cases this was incomplete in regard to lipid levels. Since detailed knowledge of lipid levels is key to fully applying lipid management guidelines [22-23], this made it difficult to provide a specific recommendation to all participants. This may have reduced the perceived value of the website, but once again the absence of full information reflects the real-world setting in which such websites operate. Finally, in regard to outcome assessment for the trial, lipid data were collected opportunistically and were incomplete, raising uncertainty about that component of the study outcomes. Nonetheless, the completeness of data for the primary outcome and many of the other secondary outcomes was good, with a strong likelihood that most participants would have been able to report reliably about these other fairly objective measures.

In conclusion, this large, carefully conducted trial found no clear beneficial health effects from a website that was designed to incorporate some of the current features of Internet-based interactive health communications applications targeted at consumers. The evidence provided here serves to again highlight the need for the comprehensive evaluation of all new strategies designed to improve population well-being if maximum value is to be extracted from the health care dollar.

## Multimedia Appendix 1

Questionnaire 1

## Multimedia Appendix 2

Questionnaire 2

## Abbreviations

BMI: body mass index
I-CAT: Internet-based Cholesterol Assessment Trial
HDL: high-density lipoprotein
LDL: low-density lipoprotein
