# Supplementary material for: Effects Of Internet-Based Tailored Advice on the Use of Cholesterol-Lowering Interventions: A Randomized Controlled Trial
Source: J Med Internet Res. 2010 Sep 13;12(3):e42. doi: 10.2196/jmir.1364 (PMC2956332; doi:10.2196/jmir.1364)
Supplement: Supplementary file 1 [file jmir_v12i3e42_app1.pdf]

THE GEORGE INSTITUTE for International Health

QUESTIONNAIRE 1

Internet-based Cholesterol Assessment Trial (I-CAT)

Thanks for agreeing to take part in this project. We now need to know a bit more information about you and your family. You may not know the answer to some of the questions but the more information you are able to provide the better so please answer as many questions as possible. It should only take you a couple of minutes. Remember, your information will only go to the researchers and will not be released to anyone else.

1. Has a doctor ever told you that you have high cholesterol?
   yes *no*
   Y N

2. Has a doctor ever told you that you have diabetes (diabetes mellitus)?
   yes *no*
   Y N

3. Has a doctor ever told you that you have high blood pressure (hypertension)?
   yes *no*
   Y N

4. Do you currently smoke cigarettes, a pipe or cigars?
   yes *no*
   Y N

5. Have you ever had a heart attack, been diagnosed with angina or had an operation of any type to improve the blood flow to your heart (such as coronary artery bypass grafting, angioplasty or stenting)?
   yes *no*
   Y N

6. If yes How old were you when you had your first heart problem?
   |__|__|__| years

7. Do you get pain in your legs that a doctor has told you is due to blocked arteries?
   yes *no*
   Y N

8. Have you ever had a stroke or transient ischaemic attack (mini stroke)?
   yes *no*
   Y N

9. Has a doctor ever told you that you have the genetic condition Familial Hypercholesterolaemia (FH)?
   yes *no*
   Y N

10. Has a doctor ever told you or any of your close relatives (parents, brothers or sisters) that you/they have a genetic condition that causes high cholesterol levels?
    yes *no*
    Y N

11. To the best of your knowledge, have any of your close relatives (parents, brothers or sisters) suffered from coronary heart disease (angina or a heart attack) at age 60 or less?
    yes *no*
    Y N

Please complete the remaining questions on the back of the sheet.

12. Are you female and post-menopausal?

yes *no*

Y N

13. How much do you weigh?

|__|__|__| Kgs or stone and pounds (please circle which unit of measurement)

14. How tall are you?

|__|__|__| cms or feet and inches (please circle which unit of measurement)

15. What is the **highest** that your total cholesterol level has ever been? mmol/l or mg/dl

|__|__|__| mmol/l or mg/dl (please circle which unit of measurement)

16. What was your **most recent** total cholesterol measurement?

|__|__|__| mmol/l or mg/dl (please circle which unit of measurement)

17. Do you know more details about your most recent levels of the different types of cholesterol (such as HDL – the good cholesterol; LDL – the bad cholesterol; and triglycerides)?

yes *no*

Y N

- *If No, skip to Q21*
- If yes, Please put in the measurements you know

18. -Low density lipoprotein cholesterol (LDL) mmol/l (in these units, usually a number less than 20) or mg/dl (in these units, usually a number more than 50)

|__|__|__|

19. -High density lipoprotein cholesterol (HDL) mmol/l (in these units, usually a number less than 5) or mg/dl (in these units, usually a number more than 15)

|__|__|__|

20. -Triglycerides mmol/l (in these units, usually a number less than 20) or mg/dl (in these units, usually a number more than 40)

|__|__|__|

21. Are you currently doing anything to try and improve your cholesterol levels

yes *no*

Y N

- *If No, you have finished*
- If yes 'What are you doing?'

22. -Eating a healthy diet (e.g. low fat and/or high fibre)

yes *no*

Y N

23. -Trying to lose weight

yes *no*

Y N

24. -Taking regular exercise

yes *no*

Y N

25. -Using special cholesterol-lowering margarine (such as ProActiv or Logichol)

yes *no*

Y N

26. -Taking tablets prescribed by my doctor

yes *no*

Y N

Thank you for completing the questionnaire.
