# Supplementary material for: Effects Of Internet-Based Tailored Advice on the Use of Cholesterol-Lowering Interventions: A Randomized Controlled Trial
Source: J Med Internet Res. 2010 Sep 13;12(3):e42. doi: 10.2196/jmir.1364 (PMC2956332; doi:10.2196/jmir.1364)
Supplement: Supplementary file 2 [file jmir_v12i3e42_app2.pdf]

INTERNET-BASED **i-CAT** CHOLESTEROL ASSESSMENT TRIAL

*Internet-Based Cholesterol Assessment Trial*

# FOLLOW-UP QUESTIONNAIRE

**The first 4 questions are about things you might have done since you completed the last questionnaire on line.**

yes no

1. ☐ ☐ Have you discussed your cholesterol levels with a health professional (e.g. doctor, nurse, dietician)?

If yes,
2. ☐ ☐ Have you started taking cholesterol-lowering medication, prescribed by your doctor?
3. ☐ ☐ Have you increased the dose of a cholesterol lowering medication you were already prescribed by your doctor?

yes no

4. ☐ ☐ Have you had your blood cholesterol checked?

**The next five questions (5 to 9) relate to your last cholesterol measurement, even if this was before you started in this study.**

mmol/l mg/dl

5. ☐ OR ☐ What was your most recent total cholesterol measurement? Even if you have not had your cholesterol checked since you completed the last questionnaire, please enter the most recent results that you have. *Please be careful to use the correct units as it may affect any advice you receive.*

yes no

6. ☐ ☐ Do you know more details about your most recent levels of the different types of cholesterol (HDL - the good cholesterol; LDL - the bad cholesterol; and triglycerides)?

mmol/l mg/dl

If yes,
7. ☐ OR ☐ Low density lipoprotein cholesterol (LDL) mmol/l (in these units, usually a number less than 20) or mg/dl (in these units, usually a number more than 50)
8. ☐ OR ☐ High density lipoprotein cholesterol (HDL) mmol/l (in these units, usually a number less than 5) or mg/dl (in these units, usually a number more than 15)
9. ☐ OR ☐ Triglycerides mmol/l (in these units, usually a number less than 20) or mg/dl (in these units, usually a number more than 40)

**The rest of the questions (10 to 26) are about things you might have done since you completed the last questionnaire on line.**

yes no

10. ☐ ☐ Has a doctor diagnosed you with high cholesterol?
11. ☐ ☐ Has a doctor diagnosed you with the genetic condition Familial Hypercholesterolaemia (FH)?

**Are you currently doing any of the following to improve your cholesterol levels?**

yes no

12. ☐ ☐ Started eating a healthy diet (e.g. low fat and/or high fibre)
13. ☐ ☐ Started trying to lose weight
14. ☐ ☐ Started taking regular exercise
15. ☐ ☐ Started using special cholesterollowering margarine (such as ProActiv or Logicol)
16. ☐ ☐ Started taking tablets for cholesterol prescribed by your doctor?

Remember, all of these questions are about things you might have done **since you completed the last questionnaire on line**.

yes no

17. ☐ ☐ Have you had your blood or urine checked for diabetes?
18. ☐ ☐ Has a doctor diagnosed you with diabetes (diabetes mellitus)?

*Continued over*

19. ☐ ☐ Have you started or increased, treatment for diabetes?

20. ☐ ☐ Have you had your blood pressure checked?

21. ☐ ☐ Has a doctor diagnosed you with high blood pressure (hypertension)?

22. ☐ ☐ Have you started or increased, treatment for high blood pressure?

23. kgs ☐ OR stone ☐ : pounds ☐ How much do you currently weigh?

24. yes ☐ no ☐ Do you currently smoke cigarettes, a pipe or cigars?

25. ☐ How many friends have you recommended this Website to? (enter 0 if none)

26. ☐ How many relatives have you recommended this Website to? (enter 0 if none)

## Supplementary Questions

**Please confirm your previous answers to these risk factors so that we can provide you with relevant advice.**

1. yes ☐ no ☐ Have you ever had a heart attack, been diagnosed with angina or had an operation of any type to improve the blood flow to your heart (such as coronary artery bypass grafting, angioplasty or stenting)?

   2. ☐ If yes, how old were you when you had your first heart problem?

3. yes ☐ no ☐ Do you get pain in your legs that a doctor has told is due to blocked arteries?

4. ☐ ☐ Have you ever had a stroke or transient ischaemic attack (mini stroke)?

5. ☐ ☐ To the best of your knowledge, have any of your close relatives (parents, brothers or sisters) suffered from coronary heart disease (angina or a heart attack) at age 60 or less?

6. ☐ ☐ Has a doctor ever told you or any of your close relatives (parents, brothers or sisters) that you/they have a genetic condition that causes high cholesterol levels?
